# Perceived Support and Sense of Social Belonging in Young Adults Who Have a Parent With a Mental Illness

**DOI:** 10.3389/fpsyt.2021.793344

**Published:** 2022-01-13

**Authors:** Aude Villatte, Geneviève Piché, Sylvie Benjamin

**Affiliations:** ^1^Laboratory LaPProche, Department of psychoeducation and psychology, Universite du Quebec en Outaouais, Saint-Jerome, QC, Canada; ^2^Centre de recherche universitaire sur les jeunes et les familles (CRUJEF), Quebec, QC, Canada; ^3^Réseau de recherche en santé des populations du Québec (RRSPQ), Montreal, QC, Canada; ^4^Réseau Intersectoriel de Recherche en Santé de l'Université du Québec (RISUQ), Quebec, QC, Canada

**Keywords:** young adults, parent with a mental illness, social support, social belonging, young carers

## Abstract

This participatory action research explores the perceived social support of youth whose parents have a mental illness during their transition to adulthood. Social support is an important protection factor during this developmental period, but few studies have explored how these young adults perceive their social support. Nor has any study assessed whether participation in a group-based participatory action research project could improve these youth's sense of support.

**Purpose:** (1) identify which aspects of social support these youth spontaneously address when talking about their experiences in Photovoice workshops; (2) explore how participants view these types of workshops as a good way to improve their sense of social support and belonging.

**Methodology:** Ten young adults (nine women and one man) between the ages of 18 and 25 who have at least one parent with a mental illness participated in *Photovoice* meetings in 2019. These group meetings aimed to explore and share their experiences as young adults whose parents have a mental illness. The testimonies were combined with data obtained from the abbreviated version of the *Social Provisions Scale* and the *Scale of Social Belonging*.

**Results:** The quantitative results suggest that participants consider their social support levels to be high, but their qualitative statements highlight low level or absence of parental support in terms of emotional, informative or instrumental levels. They see themselves as an important source of support for their parent and discuss the importance of having other supports figures (romantic partner, employer, friends, sibling, etc.). Conversely, they have difficulty asking for help for various reasons (including fear of stigma). They consider that their participation in this *Photovoice* project allowed them to feel heard, supported and to develop a sense of belonging to a group.

**Discussion:** To conclude, clinical issues to be considered for psychosocial intervention with young adults of parents with a mental illness are discussed.

## Introduction

The transition from adolescence to adulthood is a pivotal period of development occurring approximately between the ages of 18 and 25 (1), sometimes as early as age 16 (2, 3). This period is recognized as being fraught with many significant challenges (e.g., important choices to be made, autonomy to be acquired, professional domain or post-secondary education to be discovered, maintenance or adoption of healthy lifestyle habits, etc.) (1, 4, 5). Because of these challenges, this transition is particularly conducive to the emergence or worsening of mental health problems (6–8) that could then impact the entire adult life (9, 10). Findings from several studies suggest that the transition to adulthood may be particularly challenging for youth who have a parent with a mental disorder, who represent 12 to 37% of youth (11–13). Compared to their peers, these youth are more likely to have a mental illness, report more psychological distress, both internalizing and externalizing symptoms, feelings of isolation and powerlessness, relationship, academic, and professional difficulties, substance use problems, and delinquent behavior (14–22). Knowing that unaddressed difficulties at this age of life could jeopardize the entire adult trajectory, in addition to generating significant economic and human costs (23), it is urgent to identify the levers likely to promote a successful transition to adulthood for these youth.

### Social Support as a Determining Variable During the Transition to Adulthood

For young people in the general population, informal (e.g., parental availability and supervision, friendships, presence of meaningful adults) and formal (e.g., program, community, policies, recreation, meaningful stakeholders) social support is one of the key predictors of the ability to cope with the challenges of transitioning to adulthood (2, 6, 10, 24–26). In particular, social support promotes the establishment of stable relationships and improves school perseverance during this period (10, 27).

Perceived social support, which refers to a person's belief in and evaluation of their connections with others, is particularly important. One meta-analysis has highlighted the link between youth's perceived social support and well-being (28), while another has exposed an inverse link between perceived social support and depressive symptoms in youth (29). Also, results from both reviews have suggested that the quality of social support is more strongly associated with well-being and depression than the size of the support network. Similarly, results of five longitudinal studies have found that social support acts as a protective factor against depression in youth during their transition to adulthood (26, 30–33). Notably, in Scardera et al. (26) longitudinal study of 1,174 young adults, those who perceived high levels of social support at 19 years old were less likely to report mental health problems such as depressive and anxiety symptoms, at 20 years old.

The social support network of young people transitioning to adulthood, in addition to being crucial for their psychosocial adaptation, undergoes profound transformations during this period. Young people going through this developmental period face major changes in their social interactions and new challenges, such as the development of autonomy toward their parents and the emergence of stability and intimacy in their social and romantic relationships (32). To varying degrees across youth in this age group (25), parental support tends to decline while peer and romantic partner support increases (34). The relative influence of parental vs. peer or romantic partner support is still a source of debate in the scientific literature. Findings from several studies have suggested that parental support is a central protective factor in the transition to adulthood (6, 25, 29), particularly in reducing depressive symptoms (35). Instead, other studies have highlighted the role of peers, explaining that they can, in some cases, mitigate more difficult family dynamics or even compensate for poor parental support (36). Specifically, at this age, youth tend to associate more closely with people who share their interests (e.g., sports, community, peer group, volunteering) and with whom they feel a connection (37). Social interactions can greatly influence their sense of social belonging, through regular encounters with their friend group, the affects generated (38), the ability for the individual to name their expectations and fears, and the development of a shared language (39). The role of the romantic partner should also be a factor to consider, at a time when love life is gradually becoming more important to many youth (36, 40). In particular, the emerging young adult gradually perceives their romantic relationship to be more reliable. A shared intimacy accentuates mutual support, and their partner provides a significant contribution by meeting various needs (36). For many young people transitioning to adulthood, their romantic partner becomes a primary source of support, which can strengthen their resilience and reduce their stress (36).

### Social Support for Young People With a Parent With a Mental Illness

Some literature points out that the role of social support in promoting well-being among youth transitioning to adulthood may be even more significant for youth considered more vulnerable during the transition to adulthood, including those from disadvantaged backgrounds or vulnerable families (29, 41), as is the case with youth that have a parent with a mental illness (8). This idea is in line with results of studies highlighting the buffer effect of social support against the negative effects of parental mental illness on child and adolescent mental health (42–44) and, more broadly, in line with the *stress-buffer model*, which suggests that the positive effect of social support should be greater in a context of adversity or significant stress (45).

The successful transition to adulthood for youth that have a parent with a mental illness may be intimately linked to the presence of support from those around them (24, 46). The presence of a positive relationship with the parent (46) and reciprocity within that relationship (24), as well as commitment and cohesion among family members (46) seem to have an influence on the young adult's well-being and resilience. In addition, the presence of positive support from peers, significant others or competent stakeholders, could act as a cross-domain buffering by helping youth that have a parent with a mental illness cope with what they are experiencing at the family level (47). In general, friendly support could temper psychological (e.g., irrational thoughts, isolation) and emotional processes (e.g., decreases shame and fear, reassurance of one's worth), especially through the guidance and assistance of a significant peer (36, 48). Moreover, several support programs targeting these young adults and enabling them to connect with their peers, especially through professionally supervised online forums, have made it possible to offer social support (8) and have shown positive effects (49, 50). In contrast, a lack of social support (e.g., parental, friendship, romantic partner, mental health professional) is an additional vulnerability factor for youth that have a parent with a mental illness at the dawn of their transition to adulthood (24, 46).

To date, the few empirical studies that have explored how youth transiting to adulthood who have a parent with a mental illness perceive the social support they receive and offer, illustrate that they generally receive little parental support in terms of emotional, instrumental or cognitive support (14) and are also often important providers of support for their parents (46, 51). Many of these young people provide emotional, financial or even instrumental support to parents from childhood onwards, through various roles and responsibilities related to the demands of daily life. The child might then fulfill various responsibilities which become more pronounced with age, which can weaken their adaptation and lead to a process of parentification (14). This process occurs as a result of parenting gaps, but can become a burden for children of all ages, including emerging adults (14, 51). Additionally, some literature points to barriers in accessing formal resources for youth that have a parent with a mental illness of all ages (51), including the lack of knowledge among mental health professionals and parents with a mental illness regarding the impacts of parental mental illness on their child's experience, as well as their support needs (8, 52, 53). The paths of youth that have a parent with a mental illness are also negatively colored by associative stigma related to their parent's mental disorder (54). Associative stigma represents social disapproval and negative reactions toward young people due to their proximity to someone with a mental illness, often reported by youth that have a parent with a mental illness (55) and hampering their willingness to seek informal or formal support (54).

In addition to being few in number, studies that have considered the perspectives of youth of parents with a mental illness transitioning to adulthood on the social support they provide and receive all adopt “traditional” research designs by interviewing youth using questionnaires or interviews, with the limitation of restricting their responses by pre-established questions. The value of using participatory research methods that focus on artistic mediums-such as the Photovoice method that relies on photography and storytelling (56)-has been underlined with other clienteles, particularly in the mental health field (57, 58). Participating in this type of research stimulates participants' reflections and expression while allowing them to become more aware of the recurring issues they encounter, to consider solutions that make sense collectively, and to feel that they are contributing to social change by producing data that will be brought to the attention of decision-makers. Furthermore, no research has evaluated the extent to which participating in a group action research project can improve the perceived social support of these youth. This question is of major interest given that this type of project could be put in place as part of prevention and intervention programs for youths that have a parent with a mental illness.

## Objectives

The present study pursues two main objectives: (1) identify which aspects of social support youth whose parents have a mental illness during their transition to adulthood spontaneously address when talking about their experiences in Photovoice workshops; (2) explore how participants view these types of workshops as a good way to improve their sense of social support and belonging. As the results of studies that have highlighted the relevance of using the Photovoice methodology with emerging adults (58), it is possible to formulate the hypothesis that proposing a Photovoice project to children of parents with a mental illness transiting into adulthood could have a beneficial effect on their feeling of belonging to a group and of feeling recognized, valued and supported.

In the present study, social support is defined as a multidimensional construct, which corresponds to a person's perception of caring or helping behaviors from people in their network (45). These behaviors can be categorized into several dimensions, including emotional (e.g., affection, empathy), instrumental (e.g., transportation) and informational (e.g., counseling) support (59). The present study will focus on self-reported perceptions of social support.

## Methods

This study is based on a secondary analysis of data from a participatory action research conducted with youth whose parents have a mental illness, using a Photovoice approach. This initial study, conducted in 2019, aimed to: (a) explore the challenges faced by youth living in such a family context during their transition to adulthood; (b) co-create with participants a recommendations report and tools that could support the transition to adulthood of youth living with a parent with a mental illness; (c) identify the benefits of participating in such a project, from the perspective of the participants themselves. It was conducted using a concurrent nested design (60) mainly focused on qualitative data (oral and written testimonies of the participants during the Photovoice meetings) and considering certain quantitative data to complete the portrait produced.

To reach the objectives of the present study, all qualitative and quantitative data shared by participants during the initial study that were specifically related to the theme of social support (e.g., group sharing around the issue of social support, evaluation of benefits in terms of perceived social support) were extracted from the initial corpus and have been analyzed. The next subsections present the methodology of the initial study and describe how the social support data were collected, specifically.

### Recruitment Procedures

A promotional poster was installed in strategic locations of two targeted regions (Gatineau, Saint-Jerome, in Quebec, Canada). Also, the advertisement was distributed widely through the mailing lists of educational institutions and community mental health organizations, as well as on various social media groups and pages. Interested potential participants were directed to the website of the research laboratory. After viewing a detailed presentation of the project, they could complete the information and consent form. This online registration was a preamble to a telephone interview which validated the eligibility as well as the informed consent of the potential participant.

### Sample

The inclusion criteria for this study were: (a) to be between 16 and 25 years of age; (b) to have a parent with mental health problems that significantly impaired their functioning in the past 18 months, as perceived by the youth; (c) that the parent's primary disorder not be substance abuse; (d) to reside within a 50 km radius of either of the two main campuses of the Université du Quebec en Outaouais (Gatineau and Saint-Jerome); and (e) to be able to speak and understand the French language.

Eighteen youth responded to the recruitment advertisement but eight of them ultimately did not follow through to meet with the research assistant for a pre-project interview or chose not to enroll in the Photovoice workshops following the interview due to a scheduling conflict. Therefore, ten participants (including nine young women) from two administrative regions of Quebec, Canada, took part in the project. It should be noted that one participant stopped coming to the meetings after the first workshop and a second stopped after the third Photovoice workshop, both for unknown reasons.

Of these ten participants, 70% lived exclusively with their parent(s), 20% had moved out of the family home and 10% stayed with their parent(s) occasionally. These young people reported a wide variability in the frequency of contact with their parents, ranging from never to several times a day. The youngest participant was 18 years old and the oldest was 25 years old (average age 21.6 years of age). The majority (90%) have brothers and/or sisters. Eighty percent of the participants were attending a post-secondary institution while also having a part-time job. In addition, half of the sample felt they were in a precarious financial situation.

All of the youth reported having a mother with a mental illness and 40% of them reported the presence of a disorder on their father's side as well (see Table 1). Reported parental mental illnesses were various: major depressive disorder, borderline personality disorder, and anxiety disorder, sometimes with comorbid substance use problems. All participants indicated that their parent's mental disorders had been present for several years.

**Table 1:** Family characteristics of participants.

	**Affected parent**	**Diagnosis**	**Chronicity**
Participant 1	Mother and father	Depressive/substance use disorder	Since about 10 years
Participant 2	Mother	Depressive/substance use disorder	Since about 7 years
Participant 3	Mother	Anxiety/depressive disorder	Since about 11 years
Participant 4	Mother	Borderline, depressive and anxiety disorders	Since about 12 years
Participant 5	Mother	No diagnosis but anxiety/depression	Since about 20 years
Participant 6	Mother and father	Anxiety/depressive disorder	Since about 11 years
Participant 7	Mother and father	Anxiety/depressive disorder	Since about 10 years
Participant 8	Mother	Anxiety disorder	Since about 7 years
Participant 9	Mother	Anxiety/bipolar disorder	Since about 10 years
Participant 10	Mother and father	No diagnosis but anxiety/depression	Since “always”

As for the mental health of the participants, 40% of them indicated that they had received a diagnosis, ranging from anxiety disorders, mood disorders or borderline personality disorder.

### Data Collection

The conduct of the Photovoice project, the number of meetings and the number of participants were planned according to the recommendations of Wang and Burris (56). The participants were divided into two groups, according to their location and the meetings were conducted in presence spanning April through June 2019. Two facilitators (one male and one female), trained in the method and supervised by the research team throughout the project, accompanied each group by providing information and stimulating participation and discussion (e.g., by inviting youth to elaborate on their comments). In the first meeting, participants were first asked to answer the pre-participation questionnaire allowing, among other things, to evaluate their level of perceived social support. Then, the facilitators introduced participants to the Photovoice methodology, to the ethical stakes of such an approach and to the theme around which they would be led to express themselves using photos. before workshops. Over the course of the four next 2-h meetings, each participant had the opportunity to present his or her photos taken to testify to the experience of having a parent with a mental disorder to the group. The group members could react to each other's photos by naming how they related to their own experiences, for example. The participants' comments during these group meetings were recorded in audio format and a verbatim transcription was made. In addition to oral comments on the photos, participants were asked to write a caption and a title summarizing the message conveyed by each of their own photos. These written texts were given to the facilitators at the end of each meeting and were transcribed. The last three meetings allowed the youth to create awareness tools for different audiences, in order to convey the major issues that emerged from their discussions (see https://lapproche.uqo.ca/projets/photovoice1625/ for an overview of the tools co-created with the participants). During the last meeting, participants were asked to respond to the post participation questionnaire (level of perceived social support and their sense of social belonging to the project). Then, 6 months later, an online questionnaire was sent to participants, concerning the perceived benefits of their participation in the Photovoice group meetings.

### Variables and Measures

#### Perceived Social Support

The verbatim excerpts and written captions of the photos addressing content related to the concept of perceived social support were combined to form the qualitative data corpus. This selection was carried out by the principal researcher and a master's student.

In order to enrich this qualitative information, the social support variable was also examined pre- and post-project using Caron's (61) abbreviated version of the *Social Provision Scale* (SPS-10). This 10-item self-report questionnaire assesses the youth's perception on five social support dimensions (Attachment, Social integration, Reassurance of worth, Reliable alliance, Guidance and Opportunity for nurturance), that correspond to the social support functions identified in Boucher and Laprise's (59) theoretical model. Thus, the SPS-10 proposes two items for each of the dimensions, for example: “I feel I am part of a group of people who share my attitudes and beliefs” (social integration); “I have people close to me who provide me with a sense of emotional security and well-being” (attachment); “There is someone with whom I could discuss important decisions that affect my life” (guidance); “I have relationships where my competence and expertise are recognized” (reassurance of worth); “There are people I can count on in case of emergency” (reliable alliance). Respondents were asked to rate themselves on each of these items using a four-point Likert-type scale ranging from Strongly Disagree (=1) to Strongly Agree (=4). The possible individual score on the SPS-10 ranges from 10 (very low social support) to 40 (very high social support), with a score of 30 and above meaning that the individual has high social support, either accessible, available, and satisfactory (62). The psychometric properties of this scale demonstrate its suitability, including excellent concurrent validity (61, 63). In addition, the internal consistency of the SPS-10 is excellent (alpha = 0.88).

#### Sense of Belonging

The sense of belonging to the project variable was documented in a post-project measurement with the acceptance subscale of the *Sense of Social Belonging Scale* (64). This subscale has 5 items, for example: “In my relations with the other participants, I felt supported,” on which the participants must position themselves using a seven-point Likert scale ranging from Don't agree at all (=1) to Very strongly agree (=7). The score obtained can vary from 5 (very low sense of social belonging) to a maximum score of 35 (very strong sense of social belonging). The questionnaire presents very satisfactory psychometric qualities (64): internal consistency is excellent (Alpha = 0.90), criterion validity is robust with scales evaluating social support (65) and temporal stability is satisfactory (ICC = 0.70).

#### Perceived Benefits of Project Participation

An online questionnaire, sent to participants 6 months after the end of the project, assessed the perceived benefits in terms of support and social belonging. The items in this questionnaire are open-ended, such as “How would you describe the climate and exchanges that took place within your group,” “What elements or factors led you to initially participate in the project?,” “What elements or factors invited you to maintain your participation?”

### Data Analyses

To respond to the first objective of the present study, Paillé and Mucchielli's (66) technique was used to make sense of the qualitative data (verbatim of the meetings and the photo captions). Hence, the PI conducted a content analysis of the transcribed data using an inductive method. As the data were read, codes were assigned to each new concept and then grouped into categories to create a thematic tree using NVivo12 software. After presentation of the coding tree to the research team and necessary adjustments, a final interpretation of the results was proposed. Descriptive analyses of the data collected during the pre-measure of the Social Provision Scale were analyzed using the Statistical Package for Social Sciences (SPSS, Standard edition 25.0). As stated by Creswell and Plano Clark (60), the combination of data provides a more accurate picture of the explored phenomenon.

To reach the second objective of the study, a content analysis was carried out based on the responses to the open-ended questions (post 6-month online questionnaire) using the method proposed by Thomas (67). Also, statistical analyses of the pre and post measure on the *Social Provision Scale*, as well as the post measure of the *Social Belonging Scale* were conducted, using SPSS 25.0. Paired sample *t*-tests were conducted to identify any changes in participant's perceived social support and frequency statistics were used to outline the participant's perception of social belonging at the end of the project.

## Results

### Participant's Perceptions of the Social Support Offered and Received

Thematic analysis of verbatims and photo captions addressing perceived social support allowed us to distinguish four main dimensions: the social support offered and received in the youth-parent relationship; the impact of this family dynamic on the youth; the importance of social support from people other than the parent; the challenges associated with seeking outside help. The following subsections describe what participants say about each of these dimensions and provide additional insight from the quantitative data obtained with the pre measure of the *Social Provision Scale*.

#### A Caregiver Role With the Parent: “Giving Without Expecting to Receive in Return”

During the group discussions, the participants addressed a major point in their journey, namely the fact that they embodied one of the main sources of support within their families, being at the same time supervisors, friends, guardians, and providers for their parents: “*Well, I call her...always...Sometimes I would clean her house completely. I would make her meals*” (Claudia^1^), “*I clearly gave all the money I made to my parents so we wouldn't lose the house*” (Daniel), “*My mother drinks a lot, so I told her 'I promise not to ask you any questions or judge you, but when you drink, you call me so I can go get you*” (M.C.). This role of support provider seemed to be adopted even outside the relationship with the parents. Participants emphasized how much they contributed to supporting their siblings, friends, or romantic partners, for example: “*With my friends, I'm the one who listens* (…) *and I take care of my brother a lot*.” (Daniel).

The participants seemed to be aware that this support was beyond what a young person is usually expected to provide to his or her parent: “*Act as a parent instead of the parent*.” (Joany); “*… As a child, you're not supposed to have that role with your parents”* (Bianca).

In addition, they point out the contrast with the little support they receive from the parent. Indeed, all of the participants mentioned the absence or low level of parental support, particularly in terms of emotional, instrumental and informative functions but also in terms of supervision: “*There are never any congratulations”* (Claudia).

“*Making my lunch, taking my bath, going to bed on time when I was little, it wasn't necessarily my mother who would tell me to do it or who would do it for me (…). I knew there was something wrong, something different about my mother, but it wasn't named, it wasn't presented to me, it wasn't explained to me*” (Marianne).

Through their comments, they expressed needs that were unmet by their parents, such as those for comfort, being listened to and feeling loved. They also pointed to the fact that they did not feel free to express themselves: “*I can't tell my mom I'm not well”* (M.C.).

According to the participants, weak parental support could be explained in part by the manifestations specific to their parent's disorder, which in turn lead to a lessening of the interactions between the young adult and their parent. A parent with a mental illness may be less emotionally available and demonstrate more anger leading to rejection*: “They can't take it, they already have enough of their own to deal with, so, you have to find someone else* (…). *When she (my mother) is like that, I can't talk to her. She just screams and cries. I have to do it on my own*” (Marie-Pier). The stigma surrounding mental health problems would also help explain why parents do not discuss their difficulties with their children: “*It's taboo, we don't talk about it*” (Marianne).

#### Impact of This Youth-Parent Dynamic: “Both Satisfying and Disappointing”

**Figure 1:**
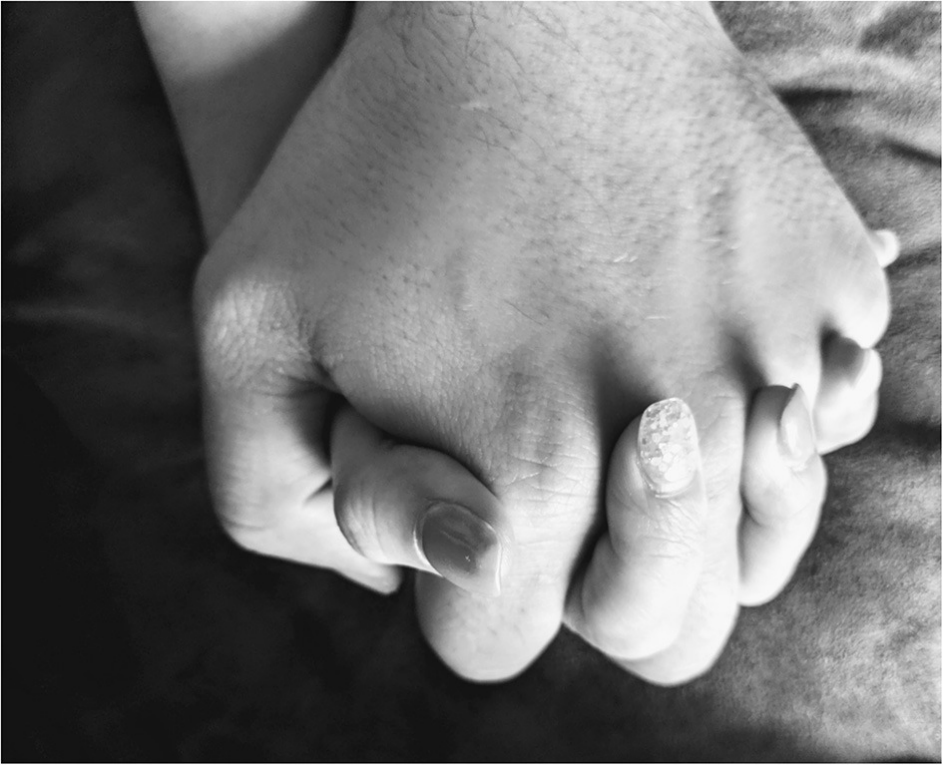
*Hand-in-Hand*: Being a caregiver for a relative with a mental disorder means offering support, encouragement and guidance without expecting to receive anything in return. It is a team effort. It means offering support, encouragement, accompaniment and help to the other person, but also forgetting oneself and risking being dragged down when the parent relapses. It also means playing the role of parent to your parent and becoming a tool for their well-being. It tinges your emotions: it's both satisfying and disappointing. But it does make you grow in any case. (Bianca).

The role of caregiver to the parent is discussed ambivalently by participants, who report both positive impacts and more negative issues about it. Figure 1 illustrates this.

Specifically, participants report a number of “positive” impacts as a result of providing strong support to their parent. The numerous tasks carried out in the perspective of supporting their parent, even their family, have contributed, according to several young people, to the development of their autonomy (e.g., ability to accomplish household tasks, set goals and maintain a budget) and social-emotional qualities (empathy, maturity, etc.) and thereby to their self-esteem: “*By always practicing this on a daily basis (supporting the parent), I consider myself a better person: more empathetic, more open to the problems of others. Sometimes I find that people are too superficial and self-centered*” (Daniel). The satisfaction of being able to help someone and the feeling of being connected to the parent were also mentioned: “*It's like being a team, doing everything together*” (Claudia).

The vocational identity of the young people also seems to be strongly influenced by their experiences as caregivers. Three of the young people in the sample chose to pursue a career path based on helping others: “…*I'm not interested in it, but it's something that's part of me too. I was born into it and it's going to follow me all my life*” (Victoria). The two participants who were already involved in a helping profession underlined the risk of overload that such a position can entail and the importance of setting limits: “*I can't intervene on what is too close to me, I can't intervene with people who have an alcohol problem*” (M.C.).

While providing support to their parents might have had some positive effects, participants noted that this role had also had significant negative repercussions. The amount of time, money and energy spent on family support may make it difficult for the young person to meet his or her own needs (e.g., thinking about what he or she would like to do with his or her life, having time to see friends.) Some participants were concerned about leaving their parents alone or leaving the family nest, while others tried to preserve the emotional well-being of their parents first, sometimes at the expense of their own well-being.

“*… I'm going to be stressed out about, like, going out at night because I know that she, she doesn't feel well. So then I start to make scenarios in my head… I'm afraid all the time… is she going to fall back into alcohol? Is she going to have suicidal thoughts because of me?... I try to comfort her in everything”*. (Marie-Pier).

These repercussions of the caregiving role can lead to cognitive and emotional fatigue, which was mentioned by six of the participants, using the following terms: “feeling worn out and old” and “really tired.” The following comments illustrate these repercussions:

“*Sometimes I tend to carry all his emotions on my shoulders, in addition to all that I'm going through... It's like I go through everything twice... there are responsibilities, that yes a child has because deep down we love our parent, but I think it can become heavy…”* (Marie-Pier).

“*… it got me down. In the sense that you know yes, you give a lot, you give a lot, but at one point it takes your energy away, it takes your time away, it brings you down”*. (Marianne).

The lack of access to information and advice can, on the other hand, generate a lot of misunderstanding and frustration toward their perception of an optimal parental support. A chain reaction can then arise, between the unspoken words, the lack of access to information, the altered communication and several negative repercussions for youth that have a parent with a mental illness (i.e., frustration, guilt, sadness, as well as psychological distress or mental health symptoms).

“*It made me angry not to understand, not knowing what was going on… and also angry toward myself, toward my mother, toward my family for not explaining it to me. It's scary to go and talk to someone, to get help”*. (Marianne).

**Figure 2:**
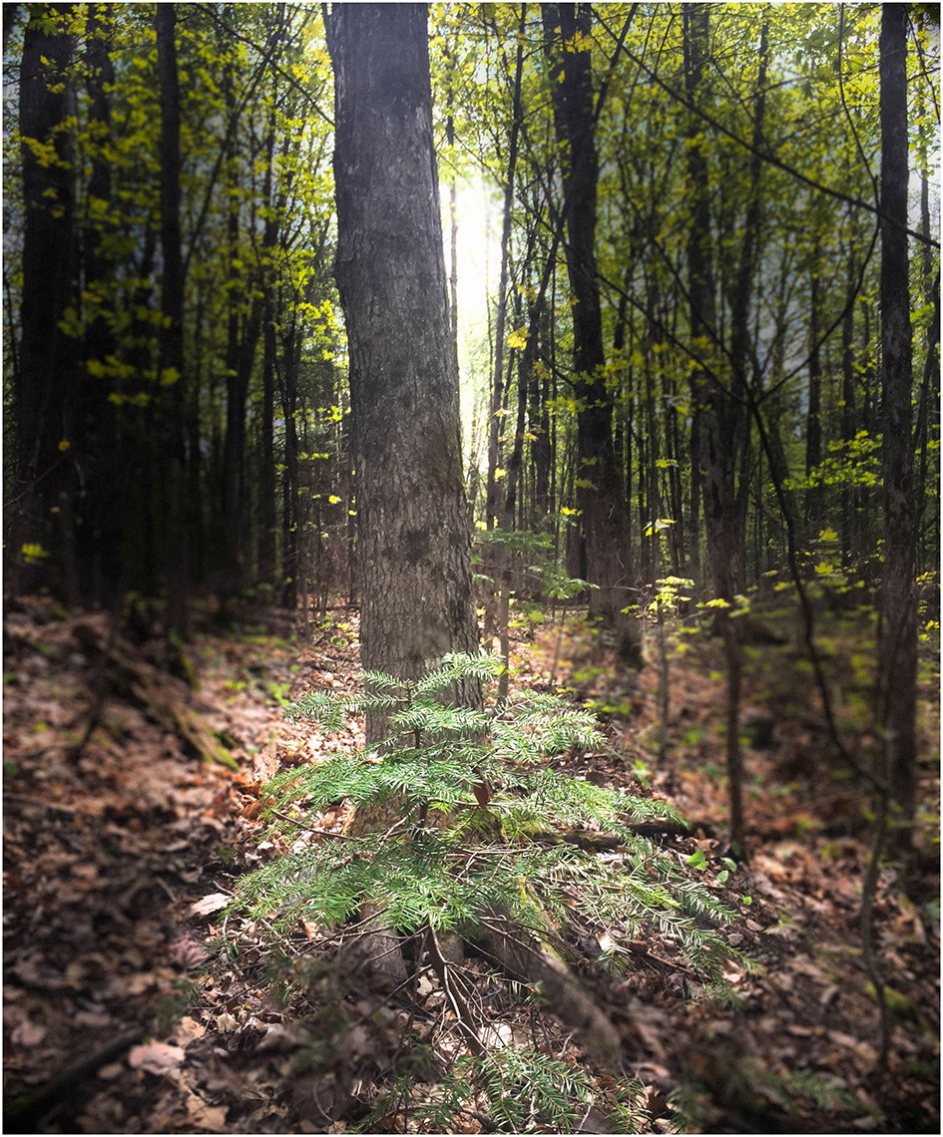
*Growing Up Small*: As we transition into adulthood, we may feel small and insecure because of the lack of role models growing up. Perhaps our role models had difficulty holding on to life themselves because of their difficulties. So, we don't feel equipped to deal with this big world and still see ourselves as small. (Victoria).

Finally, the lack of parental support may be particularly detrimental during the transition to adulthood, when young people need a positive role model to build their future adult identity (see Figure 2).

In the face of all of these impacts, many participants emphasized that limiting the support to be given to the parent and mourn the long-awaited parental support are important strategies for protecting oneself.

“*I have come to understand that you can't help someone who doesn't want to help themselves. You have to learn to understand that you can't do more than the person wants” (Bianca)*.“*At some point, in a tiredness that might have made sense, I felt compelled to set my limits. So I said to her: “You know, Mom, something really needs to happen here. This can't go on” (M.C.)*.

“*It's mourning the loss of one's parents, although you still have hope that things will get better, of course, you always have hope, but when it's continuous, consecutive through time… well, at a given moment there's this letting go, this resignation a little bit also, because of the fact that well, I don't want to have too much hope because… it's always like this*” (Daniel).

More than half of the participants mentioned that they sometimes had to distance themselves from their parent in order to refocus on themselves or to self-soothe. They attempt to recharge their batteries, as an adaptive strategy that allows them to self-regulate and let go in the face of repercussions generated by their parent's mental illness: “*I started to think more about myself than about others. I only went to see my mom on the weekends. Doing things just for me*” (Marianne).

#### Social Support as a Protective Factor: “What Helps in Dealing With the Challenges of Having a Parent With a Mental Disorder Is to Be Supported”

**Figure 3:**
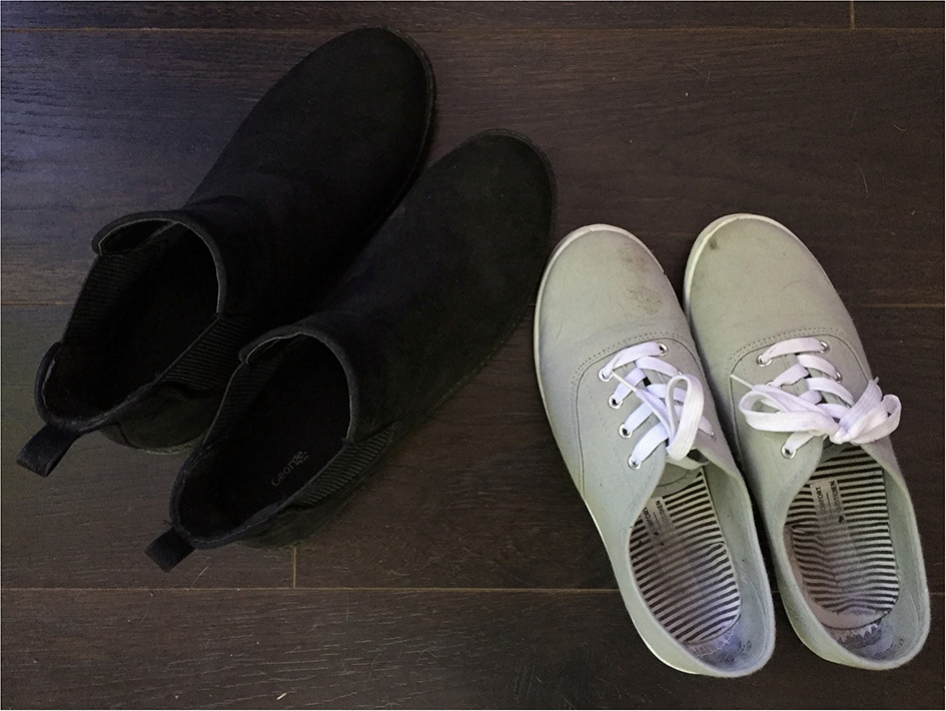
*Together*: What helps us feel better about the challenges of having a parent with a mental disorder is social support. It allows us to confide in each other during difficult times, to share our happiness and to dream together. My girlfriend and some of my friends offer me a lot of support in my daily life. (Victoria).

Having the support of one or more people around you is considered to be one of the most protective factors in dealing with the above-mentioned impacts. Victoria's words and photo (see Figure 3) testify to this.

“*You are looking out for your parent's well-being, but you are looking out for your own well-being elsewhere, by seeking support elsewhere because you are not necessarily going to get it from your parent”* (Victoria).

Almost all participants (except one) mentioned the presence of other people in their environment who played a major role in their lives and quantitative data indicate that they report a social support score that is considered high (62). In the original sample (*n* = 10), the mean score on the *Social Provisions Scale* (pre measure) was 35.2 (26.00–40.00). Only one participant fell below the threshold of 30 with a score of 26. This result is consistent with results of studies conducted on samples of emerging adults from the general population, which highlight that 10% of the sample typically does not meet the threshold for high social support (62). The results show that the dimension of social integration (e.g., “There are people who enjoy the same social activities as I do”) has the lowest mean score (x = 3.25), while tangible help (e.g., “There are people I can count on to help me in times of real need”) is the dimension with the highest mean score (x = 3.7). It thus seems that these young people better perceived the help from those around them in cases of real need or emergency (items concerning reliable alliance) vs. from participating in social activities or being part of a group (items concerning social integration). Table 2 shows the average score obtained on the SPS-10 by dimension in the pre-project measure.

**Table 2:** Social provision scale mean score (pre-project results).

	**Subscales**					
	**Attachment**	**Reliable alliance**	**Social integration**	**Guidance and opportunity for nurturance**	**Reassurance of worth**	**Total score**
Participant 1	4	4	3.5	4	4	39
Participant 2	4	4	4	4	4	40
Participant 3	4	4	4	4	3.5	39
Participant 4	3.25	3.5	3	5	2.75	30
Participant 5	4	4	3.5	4	4	39
Participant 6	3.5	3.5	2	4	4	34
Participant 7	3	3	3	1	3	26
Participant 8	4	4	4	4	4	40
Participant 9	3.5	4	2.5	3.5	3	33
Participant 10	3	3	3	3	4	32
	x = 3.63	x= 3.7	x= 3.25	x = 3.65	x = 3.63	Mean score = 35.2

Siblings, extended family members and friends are examples of support figures mentioned. For example, one participant recounted how fortunate she was to have a strong bond with her sisters which helped her cope with and normalize daily life while facing parental mental illness. This form of sibling support endured, and her strong positive assessment of it referred to mutual feedback and to the development of her own self-esteem. In the same vein, the notions of non-judgment and recognition were valued and raised when it came to proximal relationships:

“*My uncles were there and they encouraged me more than my mother. And when they saw me… “Congratulations, we are proud of you!”*” (Bianca).

The period of transition to adulthood, with the opportunities for encounters that it offers, allows the appearance of new support figures. The discussions highlighted that romantic partners become an important resource in terms of social support. Six participants explained that their romantic partners fulfilled many functions and that the latter enabled them to develop in a healthier way. This type of social support responded to all their support needs, such as emotional, instrumental, and informational. For example, participants indicated that their romantic partners “*have their back*” (comforting, loving and being loved), that part of their success was “*because of their partner*” (advice, guidance, acquiring skills), that he or she helped them overcome “*obstacles*” (meeting the demands of everyday life or dealing with problems) and that he or she contributed to their self-esteem. The great importance given to their romantic partners was mentioned several times during the meetings: “*Stability within my relationship is something… as unstable as my life has been, it's the opposite for my couple*.” (Bianca).

“*And I'm proud of what I am, vs. what I could have been if, for example, my boyfriend had not been in my life, if he had not advised me”*. (Joany).

In-laws and employers can also act as new support figures during this period: “*It's been about two years since I've started having the impression that I have a role model in my life and it is my boss who is very warm and very maternal too”*. (Victoria).

#### Challenges Encountered in the Search for External Support: “It's Not Always Easy to Get Help”

Participants in this study mentioned that the support of people close to them was sometimes no longer sufficient to meet their needs. The dimension in which they seem to consider the support of their relatives as sometimes insufficient concerns the space they would need to be able to talk about what they are experiencing with their parent. The participants emphasize that talking about what they are experiencing with their parent could be a good coping strategy (“*It helps a lot to be able to talk about it*,” Claudia), but indicate that they rarely find people to whom they can talk about the parental mental disorder.

Participants highlighted the impact the lack of information and the stigma surrounding mental health issues can have on the support they received. Notably, 80% of participants felt that most people did not understand their situation, are uncomfortable hearing what the youth has to say or would be judgmental of the youth and family: “*In my circle, that's how it's perceived: “Oh, when you talk about your mother... Oh, change the subject…, I never have the opportunity to talk about it. I always feel like I'm making them feel uncomfortable*” (Joany). Two participants reported never being comfortable discussing their parent's mental illness with anyone (friend, colleague, mental health professional, teacher, close family member). The fear of associative stigmatization (disapproval and negative reactions toward them) and auto-stigmatization (internalization of the stigma) were present in the discourse of participants and it is important to specify that they developed in an environment where they themselves had little or no information about mental illness: “*I'm so suspicious of people… to be told… that I'm not normal because of my parent*.” (Joany).

The feeling of being the only young person around them living with a parent with a mental disorder fuels their feelings of guilt, shame and ambivalence about seeking help and support, as raised in this excerpt:

“*You're isolated in this because you don't know that there are other people like you. You don't want to talk about it because you're afraid of other people's judgement, you're afraid that other people will tell you: we don't care”*. (Daniel).

While some named the challenge of verbalizing a request for help, other participants report that sometimes, they did not know where to turn for help:

“*When I was crying because I felt guilty... I didn't know who to cry to, I didn't know who to call... You go through your contact list three times, you don't call anyone after all because like, who am I going to tell this to”*. (Victoria).

“*It's not that I was alone. I had lots of people, but I couldn't talk to them*.” (Daniel).

These feelings of not being able to turn to others for fear of misunderstanding or fear of being stigmatized can lead to voluntary social withdrawal. The following example illustrates this point:

“*After that you isolate yourself because you don't want to tell others about it, you want to get better. Well, feel better”*. (Marianne).

Participants also mentioned that the felt emotional load (e.g., guilt, fear, doubt) sometimes became too high, forcing them to seek external professional help. Thus, by themselves, in a self-taught way and as a last resort, Five participants said that they had consulted professionals such as psychiatrists, doctors or psychologists in recent years, that is, during their transition to adulthood. Personal development and the urgency to take care of oneself were their main motivations.

“*I've tried a lot, but I'm at the point where I need to see a psychologist... which I didn't want to do at first, but now I'm at that point”*. (Marie-Pier).

Again, there are several fears that appear to be barriers to seeking formal help, including fear of being stigmatized and fear of harming the family system: “*You're afraid to go and see them (social workers), to tell them what you're going through, and then they call the Youth Protection and you leave. It ends up that you don't go see anyone*” (Bianca), “*You don't want the other students to see you coming out of the social worker office, because they know that there's something wrong with you or your family*” (Joany).

### The Perceived Effects of Their Participation in the Project on Social Support and the Feeling of Belonging

#### Perceived Effect of Their Participation on Sense of Support and Social Belonging

First, participants rated the *Photovoice* experience as positive: excellent (*n* = 3), very good (*n* = 4) and good (*n* = 1). Also, all youth say they would recommend participation in an equivalent project to all other young adults that have a parent with a mental illness.

Second, all participants spoke of the normalization felt and conveyed through the project as a result of countering their feelings of social isolation: “*It normalizes a lot to see that we are not alone*.^2^” Quickly, they recognized each other through their own experiences and, for the first time, they met young people with a similar background. In addition, several excerpts from the group discussions made it possible to identify a link between participation in meetings, the perception of social support and the feeling of belonging generated. The young adults took a look at their journey together and were unanimous in expressing the fact that they felt listened to, as evoked in the following extract: “*We needed to speak and we found this space… it's like a first source of support”*.

Moreover, an additional effect noted by the participants was that the exchanges allowed them to underline their respective resilience as well as to offer a discourse that conveyed hope.

“*It's true that this normalizes things and it takes us away from this view of our experience, which is a bit victimizing, where we feel alone in what we are going through, and that it's very sad...when we can just change our perspective”*.

Participants point out that their continued participation and mobilization are due to the enriching interactions between members, the desire to support other youth living this reality, and the positive repercussions on their sense of well-being.

“*What motivated me was that I felt I didn't have a space outside to talk about my parent's mental health issue and I thought I could find that space and availability, while building something bigger with our experience*.”

“*The group discussions allowing to understand each other and have strong emotional exchanges, introspection and awareness.”*

In addition, the collected responses highlight two important characteristics of this study that allowed the experience to be considered positive. Firstly, participants described the climate within the group as respectful, offering an atmosphere of openness and listening, which led to mutual trust between them. Second, they said that the support in their respective groups was understanding, empathetic and non-judgmental. One participant explained that her feeling of being supported allowed her to feel comfortable to name her personal challenges to the group.

#### Complementary Insights From Quantitative Data About Perceived Social Support and Sense of Belonging

The average score of participants on the *Sense of Belonging Scale* following their participation is 31.12 (25.00–35.00), which is considered high, considering that the maximum score is 35 (64). The results highlight that the item “In my relationships with other participants, I felt listened to” is the one that obtains the highest score. Moreover, all the answers are at the high end of the proposed Likert scale, which validates a high level of agreement toward the feeling of belonging generated within the group by the collective project.

Furthermore, the descriptive statistics from the pre- and post-measures with the SPS-10 reveal that the total mean score increased from 35.2 to 37 out of 40 (see Table 3), as did the mean scores for each dimension, with the exception of the reliable alliance dimension (e.g., “I have people I can count on in an emergency”). The greatest increase in the average score is granted to the dimension measuring the reassurance of worth (e.g., “I have relationships where my knowledge and competence are recognized”). The young people therefore evaluated having a high sense of social belonging within their group and their perception of social support improved following their participation in the project. The following verbatim illustrates the results obtained:

**Table 3:** Social provision scale mean score (by dimension; pre and post-project results).

**Subscales**	**Pre**	**Post**
Attachment	3.63	3.80
Reliable alliance	3.71	3.68
Social integration	3.25	3.56
Guidance and opportunity for nurturance	3.42	3.69
Reassurance of worth	3.38	3.75
Total	35.2	37.0

“*We sympathized with each other a lot, I think, with our lifestyles...Finally yes, it's true, I'm not alone. And, I have proof because we are very similar*.”

## Discussion

This participatory action research first documented how emerging adults whose parent has a mental illness talk about the social support they provide to their loved ones, as well as the support they receive from those around them. According to them, young people provide a great deal of support to their mentally ill parent, in all areas, in accordance with several previous studies (24, 51). Also, all youth rated their parental support as low and some as non-existent. This finding is consistent with the literature on children of parents with a mental illness of all ages, who report receiving little parental support (46, 55, 68, 69).

However, the comments exchanged during the meetings and the average score obtained by the participants on the *Social Provisions Scale*-compared to that reported in similar studies (62) show that they feel they receive a high level of social support. If their parents are perceived as unavailable and unapproachable to meet their needs, due to their mental health problems (e.g., fatigue, consumption, irritability), the youth participants perceive positive support coming from their broader social network: romantic partners, close or extended family members, bosses, friends, teachers or mental health professionals. All these sources of support remind one that the transition to adulthood, rich with opportunities to meet new people and to free oneself from parental supervision, is a period conducive to resilience (1, 2, 70, 71). Results thus suggests that because they are faced with low parental support, young adults of parents with a mental illness seek support elsewhere to meet their needs, especially emotional ones. Indeed, these different sources of support fulfill various functions, such as allowing young people to feel loved, recognized and encouraged, to receive advice and to express themselves. Consistent with work that highlights how critical these are to the development of young people in transition to adulthood in the general population (6, 25, 36), the young people who have a parent with a mental illness in our study considered them to be “vital” support, mitigating the impact of inadequate support from their parents. This qualitative finding, however, diverges significantly from the results of a large longitudinal study conducted among young people in transition to adulthood with a depressed parent, in which quality of social functioning was not identified as playing a significant protective role in resilience among youth with a depressed parent, in comparison with the parent-child relationship or the youth's intelligence quotient (46). More research is needed to clarify the nature and strength of the links between the perception of positive non-parental social support and the development of resilience in young adults with a parent with a mental illness.

Our results also made it possible to observe the little informational support that young people receive about parental mental illness, which is in line with the results of some previous studies (13, 51, 69), as well as their difficulties in mobilizing their support network and in seeking help (54). In fact, this study highlights that a range of factors create difficulties in seeking help or social support in general, such as the taboo surrounding parental illness, which seems to persist despite efforts made in recent years to reduce the stigma surrounding mental health problems (54). This finding is consistent with what several authors have noted about the impact of stigma (54) and developmental issues (such as the search for autonomy) (72) which complicate help-seeking during the transition to adulthood. Considering that participants say they want advice on mental health and the development of skills to better manage the family situation, and that half of them had asked for the advice of a mental health professional in recent years, it seems crucial to promote their access to informational social support and formal help regarding parental mental health (8, 69).

Finally, our results highlight that children of parents with a mental illness transiting to adulthood tend to isolate themselves on purpose, particularly in times when they encounter challenges. Indeed, many participants reported consciously distancing themselves from their parent or friends to regulate their negative emotions and protect themselves from the judgment of others. As well, some of them said that they had given so much to their parent that they perceived solitary withdrawal as a time of respite. Studies of other groups of youth facing bullying or stigmatization have shown that social withdrawal can be used as a coping strategy (73, 74), but the present study is the first to highlight this practice among young adults of parents with a mental illness. However, although in the short term this strategy allows youth to avoid stress and cut themselves off from the source of discomfort or suffering, it carries a risk of isolation and low social reciprocity (73).

Regarding the second research question related to improving perceived social support, notably through the feeling of belonging generated by participating in a common project, our results are encouraging. First, perceived social support increased for all the youth following their participation and their felt social belonging in this co-construction project turned out to be high. Also, at the 6-month post-project measurement, effects as well as social and relational gains from their participation were still mentioned. For some, it was about normalizing what they experienced or reducing their feeling of isolation, while others stated a better understanding of their relationship with the parent. Our findings are consistent with other studies from general population that have found beneficial influence of participation in participatory action research using Photovoice on emerging young adults (57, 58), particularly in terms of perceived support (75). The participatory and group-based approach seems to be a means of fostering a sense of belonging, particularly through the recognition of experiential knowledge (25, 39), and subsequently improving the perception of social support through exchanges between group members (38).

### Clinical Implications

Results of this study makes it possible to highlight certain specific clinical implications for mental health and education professionals who work with young adults of parents with a mental illness. First, it is suggested that professionals advocate for the development of resources specific to these vulnerable youth in schools, communities and care institutions, as well as promote the resources that are available to the latter, as young adults may not be aware of them. Because of the taboos that still surround mental health issues, their self-directedness in terms of seeking social support but also a tendency toward self-sufficiency specific to the transition to adulthood, these youth may have difficulty seeking help despite recognizing that it could be helpful. Recommendations for facilitating access to services and interventions are highlighted, based on the reflections of young adults of parents with mental illness who participated in a complementary component of this study (76). They suggested, among other things, greater visibility and diversity of mental health resources, adaptation of communication channels for youth their age (e.g., online, anonymous, interactive, social via networks), and explanation of rights and confidentiality rules as part of appropriate support. In addition, participants recommended that mental health and education professionals be open in their approach, advocate for an egalitarian relationship and provide a discreet environment. In particular, professionals need to consider the difficulties these youth may face, such as fear of being judged on their experiences and their lack of knowledge about parental illness due to poor communication.

Second, results of this study underline the relevance of systematically evaluating the quality of social support from various sources, both intrafamilial (parents, siblings) and extrafamilial (friends, romantic partners, colleagues, employers), as well as both informal and formal (e.g., intervener, support group) among these youths. Apart from standardized evaluation instruments, the proximity circle, an interpersonal psychotherapy (IPT) clinical tool, could be used to help youths identify important people and resources in their social network, in a more informal way (77). In addition to evaluating this aspect of the young adult's experience, results of this study, in accordance with recommendations from recent studies, advise that professionals support youth of parents with a mental illness in their social needs (78), including by helping them find strategies to maintain or improve their support network. This can be achieved through various manualized individual, group-based, or online interventions (79), or through the use of an informational booklet (80). In the same vein, it is also suggested that professionals experiment with various intervention modalities (e.g., discussion groups, digital platforms) to transmit information and offer support to young people whose parent has a mental illness, and use various mediums (e.g., photography, writing, art) to reduce the barriers to help-seeking and promote participant introspection. The development of a participatory group project may be an interesting strategy to facilitate a sense of being heard and sharing with others (peers and supportive adults). It would be beneficial to the resilience of these youth who are used to dealing with stigma and rejection within their usual peer group and who report lacking support from a parental figure (81).

In addition to providing psychosocial services and ensuring that they are accessible (e.g., by offering them online and in the environments frequented by young people on a daily basis) and visible, particularly through attractive promotion on social networks, it is important to provide a variety of concrete assistance measures (e.g., financial support, logistical assistance such as meal deliveries, academic accommodations to facilitate school-work-family balance) so that young people feel supported. The recent implementation of a Policy for Caregivers in Quebec (82), which aims to raise awareness among different audiences of what young caregivers may experience, while recognizing and valuing their role more and offering them assistance measures (e.g., respite), seems to be a promising avenue for young people who play a caregiver role to feel recognized and supported.

Support for families is also an important avenue. Improving the adult care system and providing support to parents could also relieve the youth of a sense of responsibility. Working with families to support parents in becoming more stable, in offering a secure and positive home for their children, and having a sufficient safety net around them would probably make it easier for the youth to become independent and to project themselves positively into their adult lives.

Continued efforts to reduce the stigma surrounding mental health problems and to promote positive mental health and support-seeking as an effective strategy for living a fulfilling life seems, at last, essential (76). The more young people who have parents with mental health problems are exposed to people who are aware of what they may be experiencing and the right strategies for individual and collective self-care, the more likely they are to feel supported.

### Strengths and Limits

One of the strengths of this study is that it allowed the youth participants to have an initial space to talk about what they were experiencing and to explore their reality with other young people living in similar situations. This first step brought a sense of normalization and provided a stepping stone to further define themselves and find meaning in their difficult family situations (52). By recognizing their lived experience, the Photovoice method contributes to the involvement and commitment of young adults (83). In fact, the participants stated that they would recommend participation in a similar project to all young adults of parents with a mental illness like themselves.

However, the study has some limits that need to be clarified. First, the sample size is small, which provides unrepresentative results; therefore, it is not possible to infer or generalize the results to all young adults of parents with a mental illness at the outset. The research design also does not make it possible to determine causal links. Furthermore, the scope of the results is limited since the sample is mainly composed of women and youth whose parents have anxiety-depressive disorders. On another note, some dimensions of social support were not examined in this study (59). Although the SPS-10 has excellent psychometric qualities (63), it would be beneficial to combine its use with instruments assessing the diversity and size of the network, as well as the quality of relationships (84) in order to capture the multiple facets of the concept of social support in future studies.

## Conclusion

In conclusion, this participatory action research study documented the perception of social support among young people transitioning to adulthood whose parent has a mental illness. The results highlight low levels of felt parental support, as noted by all the participants, and their great difficulty in soliciting support from their social network, even though they considered it essential, even vital. Participants pointed to the major importance of other significant people in their environment, such as their romantic partners, friends or mental health professionals, as a source of formal social support. In general, our results underline the relevance of providing young adults whose parent has a mental illness with resources that meet their needs and recognizing the specific issues related to their transition to adulthood, especially in a context where they frequently provide major support to their parent.

## Data Availability Statement

The raw data supporting the conclusions of this article will be made available by the authors, without undue reservation.

## Ethics Statement

The studies involving human participants were reviewed and approved by Comité d'Éthique de la recherche de l'Université du Québec en Outaouais (UQO) (2019-191). The patients/participants provided their written informed consent to participate in this study. Written informed consent was obtained from the individual(s) for the publication of any potentially identifiable images or data included in this article.

## Author Contributions

AV undertook and led the development of the research design and methodology, with contributions from GP. AV and SB undertook the literature review and led the data collection, with contributions from GP. SB led the data analysis and interpretation of findings, with contributions from AV and GP. SB and GP wrote the manuscript with editing/contributions from AV. All authors contributed to the article and approved the submitted version.

## Funding

Data collection for this study was funded by the Social Sciences and Humanities Research Council (CRSH 892-2018-2024).

## Conflict of Interest

The authors declare that the research was conducted in the absence of any commercial or financial relationships that could be construed as a potential conflict of interest.

## Publisher's Note

All claims expressed in this article are solely those of the authors and do not necessarily represent those of their affiliated organizations, or those of the publisher, the editors and the reviewers. Any product that may be evaluated in this article, or claim that may be made by its manufacturer, is not guaranteed or endorsed by the publisher.

## Footnotes

^1^Some participants chose to keep their first name, others chose a pseudonym.

^2^The questionnaires completed six months after participation in Photovoice meetings were completed anonymously. It is therefore not possible to identify which participant made the comment.
